# Atopic Dermatitis Accelerates Skin Physiological Functional Decline and Visible Aging, Suppressed by Skincare Habits

**DOI:** 10.1111/jocd.70707

**Published:** 2026-02-10

**Authors:** Katsuko Kikuchi, Yumi Murakami, Haruna Sato, Ryoko Yamashita, Yumiko Saya, Rikako Uchino, Takuya Sugita, Yosuke Shinkai, Yusuke Takahara, Kenta Shingaki, Hiroshi Matsunaka

**Affiliations:** ^1^ Sendai Taihaku Dermatology Clinic Sendai Miyagi Japan; ^2^ Department of Dermatology Tohoku University Graduate School of Medicine Sendai Miyagi Japan; ^3^ NOV Academic Research, TOKIWA Pharmaceutical Co. Ltd. Minato‐ku Tokyo Japan; ^4^ Groupwide Research and Development Tokyo Research Laboratory Noevir Co. Ltd. Kawasaki Kanagawa Japan

**Keywords:** atopic dermatitis, inflammatory factors, skin physiological function, skincare, stratum corneum, visible aging

## Abstract

**Background:**

Atopic dermatitis (AD) is an inflammatory skin disorder that causes barrier dysfunction and chronic itching; patients exhibit more obvious age‐related external changes than healthy individuals. Detailed comparative investigations of patients with AD and healthy individuals are lacking, including visible age, skin physiological function, and inflammatory and immune factors in the stratum corneum.

**Aims:**

To investigate the skin physiological function, physiologically active substances in the stratum corneum, and visible age of patients with AD and healthy controls (HCs), and to verify the effects of skincare habits.

**Methods:**

This study was conducted from November to December 2023 and included male and female patients with AD and HCs aged 20–50 years. Skincare habits were assessed using a questionnaire, physiological function by device measurements, physiologically active substances using multiplex analysis, and visible aging by comparison with age‐ and sex‐matched patients with AD and HCs.

**Results:**

Compared with those in the HC group, the cheek skin of the AD group was darker, had a higher melanin index, lower stratum corneum water content, higher transepidermal water loss, higher levels of inflammatory and immune factors in the stratum corneum, and less sebum on the forehead skin surface. Additionally, the AD group appeared older than their age‐matched HC group. However, skincare habits improved these changes, with no significant difference between the HC and AD groups following skincare habits.

**Conclusions:**

Skincare habits can suppress AD‐associated changes in the physiological function of the skin, inflammatory and immune factors in the stratum corneum, and visible aging.

## 1 Introduction

Atopic dermatitis (AD) has an estimated prevalence of 2%–20% worldwide, varying by ethnicity and age [1]. In Japan, the estimated prevalence is 12.9% among individuals aged 6 months to 6 years, 10.3% among those aged 6–12 years, 9.1% among those aged 12–18 years, and 10.7% among individuals aged 6 months to 18 years [2]. AD is an inflammatory skin disorder that causes chronic itching, and patients with AD have dry skin with a barrier dysfunction of the epidermis [3]. This barrier dysfunction is associated with the severity of AD [4]. Additionally, patients with AD exhibit changes in the expression of various inflammatory cytokines compared with healthy individuals. Serum thymus and activation‐regulated chemokine (TARC) concentration has been shown to be associated with the severity of AD and skin barrier function, and differences in cytokine expression levels between affected and unaffected local skin sites have been reported; these molecules are therefore indicators of disease activity for AD [5, 6, 7].

Skin aging is separated into physiological aging and aging associated with environmental factors. Physiological aging is the aging of the skin that naturally takes place with increasing age. During physiological aging, the skin becomes drier and thinner, loses its elasticity, and develops fine wrinkles and pigment deposition. Aging associated with environmental factors such as ultraviolet rays, smoking, and air pollution accelerates physiological aging. Additionally, it is believed that inflammation and changes in immune function are also involved in the mechanisms of skin aging caused by these factors [8, 9, 10]. The skin of patients with AD undergoes more obvious external age‐related changes than the skin of HCs does and may therefore age more quickly. However, to date, no study has conducted a detailed comparison of patients with AD and healthy individuals in terms of factors such as visible aging, skin physiological function, and cytokine concentrations in the stratum corneum.

The treatment administered for AD consists mainly of eliminating aggravating factors and applying anti‐inflammatory topical medications such as steroids. Additionally, systemic therapies with JAK inhibitors or biologics have recently been used for moderate or worse AD [11]. Alongside these pharmacological treatments, washing, moisturizing, sunscreen, and other appropriate forms of skincare are also important in controlling skin symptoms [11, 12]. Skin surface contaminants (e.g., dead skin cells, topical agents, lipid peroxides, and external debris) can exacerbate skin conditions; therefore, they should be gently removed using a mild cleanser [13]. The application of moisturizers, such as ointments or creams containing occlusive oils, reduces transepidermal water loss (TEWL) and increases the moisture content of the stratum corneum [4, 14, 15, 16]. Because exposure to excessive nontherapeutic ultraviolet rays (sunlight) can also be an aggravating factor, avoiding sunlight by means of clothing, sunscreen, or other methods is recommended [17]. Overall, appropriate skincare can help alleviate skin symptoms and prevent flare‐ups.

The aim of this study was to investigate the effects of skincare habits on the visible age, skin physiological functions and physiologically active substances in the stratum corneum of patients with AD. Therefore, we conducted a detailed analysis of skin physiological function and physiologically active substances in the stratum corneum of the cheek skin of patients with AD and healthy controls (HCs) and assessed the visible aging that they presented with. We also investigated the association between these and skincare habits.

## 2 Materials and Methods

### 2.1 Study Design

Our non‐interventional cross‐sectional study was conducted between November and December 2023 under the supervision of a dermatologist. Male and female patients with AD and HCs (aged 20–50 years) were recruited for the study. Briefly, the subjects first underwent primary selection using an online pre‐study survey. Among these individuals, we selected those who attended an in‐person medical examination by a dermatologist upon starting this study and those who met the selection requirements for this study. We measured and compared skin physiological function, physiologically active substances in the stratum corneum of the cheek skin, and visible age between the AD and HC groups. Additionally, we made a comparison between four different groups: all patients (AD‐All), patients with mild AD (AD‐Mild), patients with moderate or worse AD (AD‐Over moderate), and the HC group. Moreover, we investigated the effects of daily skincare habits on the skin parameters, including skin appearance.

This study was approved by the Yoyogi Mental Clinic Research Ethics Committee (approval number TKW312). A detailed explanation of the experimental procedure was provided to each subject before obtaining the signed consent form.

### 2.2 Participants

Male and female patients with AD and HCs aged between 20 and 50 years were included. The AD group comprised subjects who had suffered from AD since infancy or childhood, and whose symptoms had been stabilized with treatment (not currently undergoing systemic therapy). Additionally, the subjects were patients whose general level of severity was mild or worse according to the Japanese Society of Allergology Atopic Dermatitis Treatment Guidelines 2024 [11], with severity score ≤ 3 (0: none, 1: slight, 2: mild, 3: moderate, 4: severe) according to a five‐point scale for facial skin findings (erythema, edema/papule, percolate/scabs, abrasion marks, and lichenization), and who did not require topical therapy. In contrast, the HCs were individuals who had never been diagnosed with AD and had no other skin disorders or cosmetic allergies.

Potential participants were excluded if they met any of the following criteria: spending ≥ 4 h per day outdoors without measures to avoid sunburn (to exclude individuals with extreme sun exposure) or having a body mass index ≥ 25 kg/m^2^ (to reduce bias when comparing visible aging).

### 2.3 Physiological Functioning of the Skin (Skin Measurements)

Before any measurements were made, the participant cleaned the face with a cleanser and facial wash and rested for 15 min in a room maintained at an ambient temperature of 22°C ± 2°C and humidity of 50% ± 5%. Skin color was measured once on the right cheek with Antera 3D (Miravex Limited, Dublin, Ireland). The amount of sebum on the skin surface was measured twice on the forehead using a Sebumeter SM 815 (Courage+Khazaka Electronic GmbH, Cologne, Germany), and TEWL was measured twice on each of the left and right cheeks using a Tewameter TM HEX (Courage+Khazaka Electronic GmbH). Skin pH was measured with a Skin‐pH‐Meter PH 905 (Courage+Khazaka Electronic GmbH), and the melanin and erythema index values were measured with a Mexameter MX 18 (Courage+Khazaka Electronic GmbH). The stratum corneum water content was measured five times each on the left and right cheeks with a SKICON‐200EX (Yayoi Co. Ltd., Tokyo, Japan). For all measurements, the mean values were used in the analysis. If a rash or other symptoms were present at the measurement site, measurements were made to avoid the lesion.

### 2.4 Sampling of the Stratum Corneum

After skin measurements were made, stratum corneum samples were collected using the previously reported tape stripping method [5, 6]. Pieces of Cellotape (Nichiban Corporation, Tokyo, Japan), 24 mm wide and 60 mm long, were applied to the left and right cheeks, gently pressed down, and then peeled off to obtain the stratum corneum. Sampling was conducted three times each (three layers) on both cheeks. The collected stratum corneum was stored on the adhesive tape at −20°C until analysis. To improve consistency between participants, the first of the three layers was discarded, the second layer was used as the measurement sample, and the third layer was kept as a spare sample.

### 2.5 Physiologically Active Substances in the Stratum Corneum (Multiplex Analysis)

The tape with the stratum corneum attached was cut into strips approximately 5 mm wide, immersed in phosphate‐buffered saline containing 0.05% Tween 20, and fragmented using ultrasonics on ice for 30 s. The resulting solution was filtered through an Ultrafree‐MC‐GV Durapore‐PVDF 0.22 μm (MilliporeSigma, Burlington, MA, USA) and concentrated with an Amicon Ultra‐0.5 Ultracel‐3 (MilliporeSigma) for use in measurements. A Bio‐Plex 200 (Bio‐Rad, Hercules, CA, USA) was used to obtain the measurements, and Bio‐Plex Manager 5.0 software (Bio‐Rad) was used for the analysis. All operations were performed according to the manufacturer's instructions.

A Luminex assay kit (R&D Systems Inc., Minneapolis, USA) was used to measure 16 factors, namely, interleukin 4 (IL‐4), IL‐8, IL‐10, IL‐13, IL‐34, macrophage migration inhibitory factor (MIF), C‐X‐C motif chemokine ligand 10 (CXCL10), monocyte chemotactic protein‐1 (MCP‐1), TARC, granulocyte‐macrophage colony‐stimulating factor (GM‐CSF), matrix metalloproteinase‐1 (MMP‐1), matrix metalloproteinase‐2 (MMP‐2), matrix metalloproteinase‐9 (MMP‐9), receptor for advanced glycation end product (RAGE), vascular endothelial growth factor A (VEGF‐A), and pentraxin 3 (PTX3). Protein levels were measured using Pierce BCA Protein Assay Kits (Thermo Fisher Scientific Inc., Waltham, MA, USA). The analysis values obtained by multiplex analysis were corrected for protein levels to use in the analysis.

### 2.6 Visible Aging and Average Facial Images

After face washing and before skin measurements were made, each participant's face was photographed with a Vectra Handy H2 (Canfield Scientific Inc., Parsippany, NJ, USA). Participants were divided into groups by age and sex: male patients and HCs aged 20–34; female patients and HCs aged 20–34; male patients and HCs aged 35–50; and female patients and HCs aged 35–50. A relatively youthful appearance score was computed based on pairwise comparisons; the appearance youthfulness of the members of each group was compared with that of the other members of the same group. Eleven assessors placed photographs of two members of the same group side by side and chose the one they considered looked younger; this participant was given a score of 1 point. This procedure was followed for all the combinations of participants within the group. The total score for each participant was calculated as the sum of the scores (from 0 to (number of participants in the group － 1) × 11 (number of assessors)). The resulting number was divided by 11 (the number of assessors) to obtain each participant's pairwise youthfulness score. The 11 assessors were chosen from among employees of the research laboratory who were not involved in this study, and there was no gender or age bias.

Average facial images of eight groups (male patients aged 20–34, male HCs aged 20–34, female patients aged 20–34, female HCs aged 20–34, male patients aged 35–50, male HCs aged 35–50, female patients aged 35–50, and female HCs aged 35–50) were generated from facial photographs using HBM Rugle software (Medic Engineering Corporation, Kyoto, Japan).

### 2.7 Frequency of Everyday Skincare

Patients were scored on their facial skincare habits based on their answers to a questionnaire. Scores were assigned for the weekly frequency of use (daily: 5 points; 5–6 times/week: 4 points; 3–4 times/week: 3 points; once or twice a week: 2 points; never: 1 point) and the number of uses a day (at least twice a day, morning and evening: 3 points; either morning or evening: 2 points; never: 1 point) of each of the following items: facial cleanser, cleansing product, moisturizing lotion, serum, emulsion, cream, and sunscreen. The frequency‐of‐use score and the daily‐use score for each skincare item were multiplied, and the resulting values were summed to obtain a total skincare habit score (range, 7–105) across seven items. Thereafter, patients were divided into groups of those with higher (AD‐Frequent skincare) and lower (AD‐Infrequent skincare) scores than the median score for all patients. The measured values and assessments of the patients were then compared with those of the HC group. Separately, patients were also divided into those who used sunscreen (AD‐Sunscreen) and those who did not (AD‐No‐Sunscreen); the values for these groups were similarly compared with those of the HC group.

### 2.8 Statistical Analysis

All statistical analyses were performed using SPSS Statistics (Ver. 30; International Business Machines Corporation, Armonk, USA). Significant differences between groups were determined using the Mann–Whitney *U* test for two‐group comparisons and the Kruskal–Wallis test and Dunn‐Bonferroni test for three‐group comparisons. Spearman's rank correlation coefficient was used to evaluate correlations between different measurement parameters and assessments. Data are presented as a box‐and‐whisker plot or mean and standard deviation. Statistical analysis was conducted for groups with at least five measured values or scores. *p* < 0.05 was considered statistically significant.

## 3 Results

### 3.1 Participant Characteristics

The participants comprised 36 subjects diagnosed with AD by the study physician and meeting the selection criteria (16 males, aged 32.3 ± 9.2 years; 20 females, aged 32.6 ± 9.1 years) and 48 HCs (24 males, aged 32.1 ± 8.3 years; 24 females, aged 31.8 ± 8.1 years). No significant difference in age was observed between the AD and HC groups. The severity of AD was mild in 22 cases, moderate in 11, severe in 3, and most severe in 0 (Table 1). Moreover, comparisons were conducted between four different groups: all subjects (AD‐All group, *n* = 36), subjects with mild AD (AD‐Mild group, *n* = 22), subjects with moderate or worse AD (AD‐Over moderate group, *n* = 14), and the HC group (*n* = 48).

**TABLE 1:** Participants' background characteristics.

Characteristic		AD (*n* = 36)		HCs (*n* = 48)		*p*
		Male	Female	Male	Female	
Sex	Number	16	20	24	24	0.641, NS
Age (years)	Number					0.947, NS
	20–34	8	9	12	12	
	35–50	8	11	12	12	
	Mean ± SD	32.3 ± 9.2	32.6 ± 9.1	32.1 ± 8.3	31.8 ± 8.1	
Disease severity	Number					
	Mild	9	13			
	Moderate	5	6			
	Severe	2	1			
	Most severe	0	0			

*Note:* The average age is shown as mean ± SD. The *p*‐value for comparison between subjects with AD and healthy controls was calculated using the chi‐squared test. Disease severity was determined using the Japanese guidelines for atopic dermatitis 2024 [11].

Abbreviations: NS, not significant; SD, standard deviation.

### 3.2 Physiological Functioning of the Skin

**FIGURE 1:**
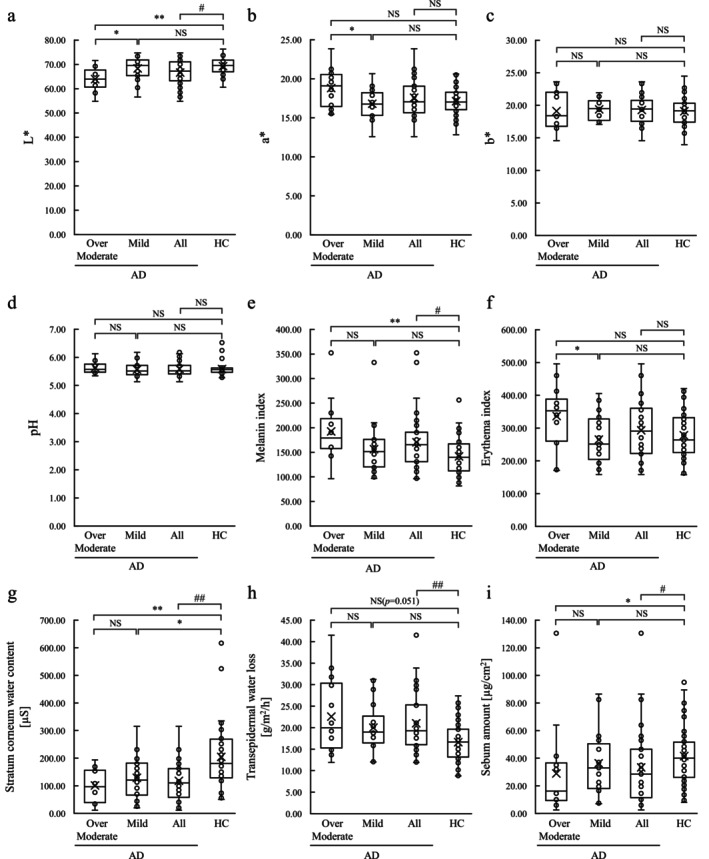
Box plots of skin physiological function in the AD and HC groups. (a–c) Skin color measurement, (d) pH, (e, f) melanin and erythema indices, (g) stratum corneum water content, (h) transepidermal water loss, and (i) sebum amount. The *p*‐value for comparing AD‐Mild (*n* = 22), AD‐Over moderate (*n* = 14), and HC groups (*n* = 48) was calculated using the Kruskal–Wallis test and Dunn‐Bonferroni test. *p < 0.05; **p < 0.01. The *p*‐value for comparing AD‐All (*n* = 36) and HC groups (*n* = 48) was calculated using the Mann–Whitney *U* test. #*p* < 0.05; ##*p* < 0.01. AD, atopic dermatitis; HC, healthy control; L*, skin lightness value; a*, skin redness value; b*, skin yellowness value; NS, not significant.

When comparing AD‐All with the HC group, AD‐All had lower lightness (L* value), a higher melanin index, lower stratum corneum water content, higher TEWL on the cheek skin, and reduced sebum on the forehead skin surface. A three‐group comparison among AD‐Mild, AD‐Over moderate, and HC groups showed that skin lightness (L* value) was higher in the AD‐Mild and HC groups, and skin redness (a* value) was lower in AD‐Mild compared with AD‐Over moderate. The melanin index of AD‐Over moderate was higher than that of the HC group, and the erythema index was also higher than that of AD‐Mild. The stratum corneum water content was lower, and the amount of sebum on the skin surface was less in the AD‐Over moderate group than in the HC group. AD‐Mild also had lower stratum corneum water content than the HC group (Figure 1a–i).

### 3.3 Physiologically Active Substances in the Stratum Corneum (Multiplex Analysis)

**FIGURE 2:**
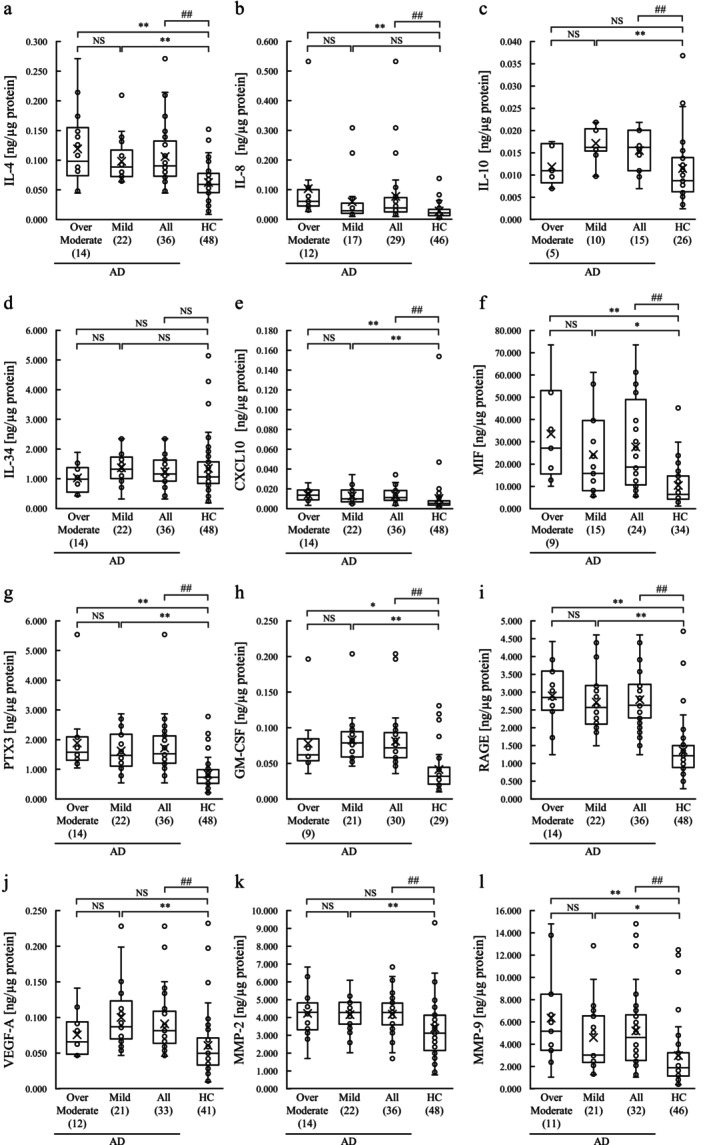
Box plots of the physiologically active substances in the stratum corneum of the AD and HC groups. The total number of samples in each group was 22 for AD‐Mild, 14 for AD‐Over moderate, 36 for AD‐All, and 48 for HC. (a) IL‐4, (b) IL‐8, (c) IL‐10, (d) IL‐34, (e) CXCL10, (f) MIF, (g) PTX3, (h) GM‐CSF, (i) RAGE, (j) VEGF‐A, (k) MMP‐2, and (l) MMP‐9. Molecules detected in fewer than five samples were not quantified. For molecules detected in five or more samples, only the detected samples were analyzed. The numbers of samples included in each analysis are indicated in the graphs. The *p*‐value for comparing AD‐Mild, AD‐Over moderate, and HC groups was calculated using the Kruskal–Wallis test followed by the Dunn‐Bonferroni test. *p < 0.05; **p < 0.01. The *p*‐value for comparing AD‐All and HC groups was calculated using the Mann–Whitney *U* test. #*p* < 0.05; ##*p* < 0.01. AD, atopic dermatitis; HC, healthy control; CXCL, C‐X‐C motif chemokine ligand; GM‐CSF, granulocyte‐macrophage colony‐stimulating factor; IL, interleukin; MIF, macrophage migration inhibitory factor; MMP, matrix metalloproteinase; PTX, pentraxin; RAGE, receptor for advanced glycation end product; VEGF, vascular endothelial growth factor; NS, not significant.

Based on multiplex analysis of the physiologically active substances in stratum corneum sampled from the cheeks, the results for the 12 physiologically active substances for which at least five analysis values within the analysis software's confidence interval in all four groups were obtained out of the 16 analyzed factors are shown in Figure 2.

A comparison between AD‐All and the HC group revealed that the levels of IL‐4, IL‐8, IL‐10, CXCL10, MIF, PTX3, GM‐CSF, RAGE, VEGF‐A, MMP‐2 and MMP‐9 were higher in AD‐All. A three‐group comparison among AD‐Mild, AD‐Over moderate, and HC groups revealed that the levels of IL‐4, IL‐8, CXCL10, MIF, PTX3, GM‐CSF, RAGE and MMP‐9 were higher in AD‐Over moderate than in the HC group. Similarly, the levels of IL‐4, IL‐10, CXCL10, MIF, PTX3, GM‐CSF, RAGE, VEGF‐A, MMP‐2 and MMP‐9 were higher in the AD‐Mild group than in the HC group.

### 3.4 Visible Aging and Average Facial Images

**FIGURE 3:**
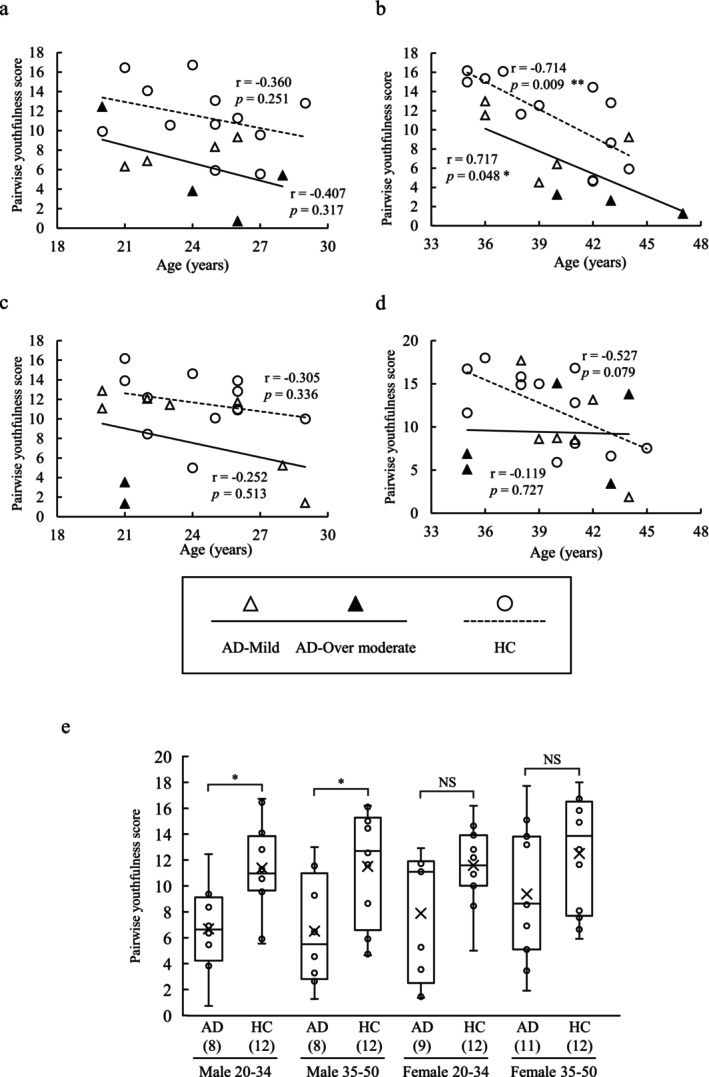
Comparison of pairwise youthfulness scores between the AD and HC groups. (a) Male participants aged 20–34 (*n* = 4, 4, 12 for AD‐Mild, AD‐Over moderate, and HC, respectively), (b) male participants aged 35–50 (*n* = 5, 3, 12), (c) female participants aged 20–34 (*n* = 7, 2, 12), (d) female participants aged 35–50 (*n* = 6, 5, 12), and (e) box plot of the pairwise youthfulness scores. The *p*‐value for comparing the AD group with the HC group was calculated using the Mann–Whitney *U* test. The number of samples is shown in each graph. *p < 0.05. AD, atopic dermatitis; HC, healthy control.

For male and female participants aged 20–34 and 35–50, the Pairwise youthfulness score tended to be lower—indicating an older visible age—for the AD group than for the HC group. Among the AD group, AD‐Over moderate tended to have lower pairwise youthfulness scores than AD‐Mild (Figure 3a–d). In the 35–50 age range, males in the AD and HC groups showed a significant age‐associated decrease in pairwise youthfulness scores (Figure 3b). The mean pairwise youthfulness score was significantly lower for the male AD group aged 20–35 and 35–50 than for their healthy counterparts; no such significant difference was evident for female participants (Figure 3e).

**FIGURE 4:**
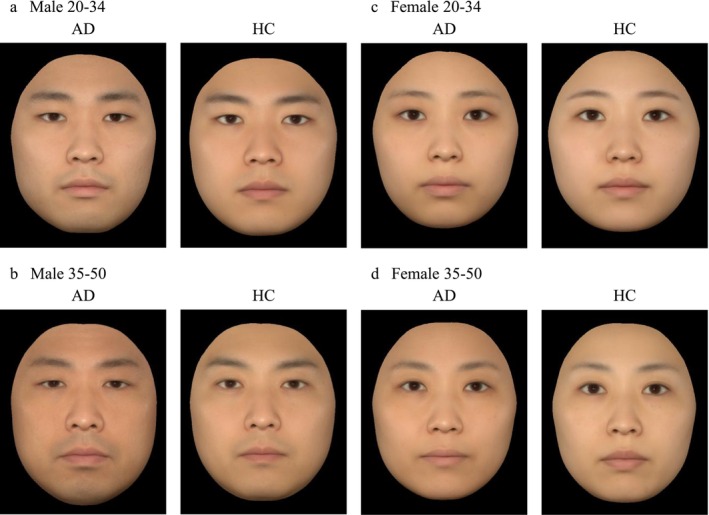
Average facial images of the AD and HC groups. (a) Male participants aged 20–34, (b) Male participants aged 35–50, (c) Female participants aged 20–34, and (d) Female participants aged 35–50. AD, atopic dermatitis; HC, healthy control.

Assessments of the average facial images did not differ greatly for male and female AD group aged 20–34 (Figure 4a,c), but for male participants aged 35–50, the average image of an individual with AD group showed darker skin and narrower palpebral fissures (Figure 4b). For female participants aged 35–50, the average image of an individual in the AD group also showed darker skin (Figure 4d).

### 3.5 Skincare Habits and Skin Condition

**FIGURE 5:**
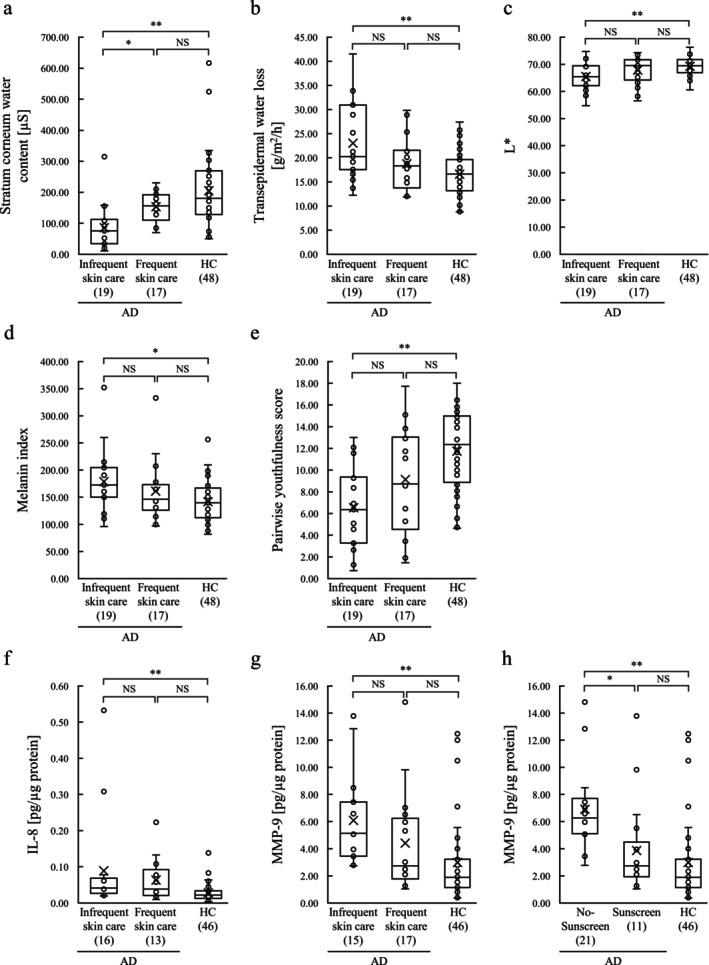
Box plots of skin properties of AD‐Frequent skincare (AD‐Sunscreen), AD‐Infrequent skincare (AD‐No‐Sunscreen), and the HC group. (a–g) Comparison of the physiological function of facial skin, physiologically active substances in the stratum corneum, and pairwise youthfulness score of AD‐Frequent skincare, AD‐Infrequent skincare, and HC group. (a) Stratum corneum water content, (b) transepidermal water loss, (c) L*, (d) melanin index, (e) pairwise youthfulness score, (f) interleukin‐8 in the stratum corneum, (g) MMP‐9 in the stratum corneum. (h) Comparison of MMP‐9 levels in the stratum corneum of AD‐Sunscreen, AD‐No‐Sunscreen, and HC groups. The total number of samples in each group was as follows: 19 for AD‐Infrequent skincare, 17 for AD‐Frequent skincare, 23 for AD‐No‐Sunscreen, 13 for AD‐Sunscreen, and 48 for HC. For graphs f, g, and h, molecules detected in fewer than five samples were not analyzed. For molecules detected in five or more samples, only the detected samples were included in the analysis. The number of samples used in each analysis is indicated in all graphs (a–h). The *p*‐value for comparing AD‐Frequent skincare (AD‐Sunscreen), AD‐Infrequent skincare (AD‐No‐Sunscreen), and the HC group was calculated using the Kruskal–Wallis test and Dunn‐Bonferroni test. The number of samples is shown in each graph. *p < 0.05; **p < 0.01. AD, atopic dermatitis; HC, healthy control; L*, lightness value; IL, interleukin; MMP, matrix metalloproteinase; NS, not significant.

Based on the score for skincare habits, subjects with AD were categorized into two groups: AD‐Frequent skincare (*n* = 17) and AD‐Infrequent skincare (*n* = 19). Compared with the HC group, the stratum corneum water content of AD‐Infrequent skincare was lower, TEWL was higher, skin color was darker, the melanin index was higher, the pairwise youthfulness score was lower, and the levels of IL‐8 and MMP‐9 in their cheek skin were higher (*p* < 0.05). No such significant differences were observed (*p* > 0.05) between the AD‐Frequent skincare and HC group (Figure 5a–g).

Based on sunscreen use, subjects with AD were categorized into AD‐No‐Sunscreen (*n* = 23) and AD‐Sunscreen (*n* = 13) groups. AD‐No‐Sunscreen had significantly higher MMP‐9 levels than AD‐Sunscreen and the HC group (Figure 5h).

## 4 Discussion

In this study, we compared the physiological function of facial skin, physiologically active substances in the stratum corneum, and visible facial aging between subjects with AD and HCs aged 20–34 and 35–50 years. Our findings showed that the skin of subjects with AD exhibited aging‐related changes. Additionally, we examined the effects of facial skincare habits on visible skin aging in subjects with AD.

In terms of the physiological function of facial skin, we demonstrated that the AD group had significantly lower stratum corneum water content and significantly higher TEWL in the cheek skin, and significantly less sebum on the forehead than the HC group did. The lightness of cheek skin was also lower, and the melanin index significantly higher in the AD group than in the HC group. Skin dryness (decreased stratum corneum water content and sebum secretion) and skin dullness (decreased lightness, increased melanin) are among the characteristics of aging skin. Therefore, our results suggest that age‐related skin changes progress more rapidly in subjects with AD than in HCs. Recent studies have highlighted dysregulation of the lipidome and defective sebaceous gland activity in adult subjects with AD. The lipid composition of sebum has been reported to be dysregulated in AD [18], and biosignatures of defective sebaceous gland activity have been observed even in sebum‐rich areas of AD skin [19]. Our results, showing significantly lower sebum levels on the forehead of AD subjects, are consistent with these findings and further suggest that sebum deficiency contributes to the overall physiological decline and dryness observed in AD facial skin.

Our analysis of physiologically active substances in the stratum corneum of the cheeks showed that levels of cytokines, chemokines, inflammation‐related proteins, and inflammatory markers were significantly higher in the AD group than in the HC group. IL‐4, IL‐8, IL‐10, and GM‐CSF are cytokines involved in immunity and allergies, whereas CXCL10, MIF, and PTX3 are chemokines, cytokines, or proteins implicated in inflammation [20, 21, 22, 23, 24, 25]. Although our study included subjects with stabilized symptoms requiring no topical therapy, our results support previous studies that the skin of subjects with AD exhibits chronic inflammation [11].

Our comparison of visible aging assessments between the AD and HC groups showed that male and female participants in the AD group tended to have lower pairwise youthfulness scores (appearing older to a third party) than those in the HC group. In particular, the scores of the male AD group in the 20–34 and 35–50 year age groups were significantly lower than those of the HC group. Among the AD group, AD‐Over moderate tended to have lower pairwise youthfulness scores than AD‐Mild. In the assessments of the average facial images, the male AD group aged 35–50 also had obviously darker skin. These external changes may be attributed to differences in skin physiological function between the AD and HC groups and the levels of the inflammation‐related factors such as IL‐8, MIF, RAGE, VEGF‐A, MMP‐2, and MMP‐9. IL‐8 is a factor in the senescence‐associated secretory phenotype (SASP) and induces cellular senescence [26]. MIF promotes skin aging by blocking peroxisome proliferator‐activated receptor (PPAR) signals, encouraging the secretion of SASP factors [27]. RAGE exacerbates oxidative stress and inflammatory response as a cell surface receptor for advanced glycation end products (AGEs) [28]. VEGF‐A increases vascular permeability, triggering inflammation [29]. MMP‐2 and MMP‐9 are gelatinases that both selectively degrade the basal membrane and decompose extracellular matrix proteins, and their expression is known to increase with age [30]. Our results suggest that these changes in physiologically active substances in the skin of subjects with AD may collectively contribute to external changes. This acceleration of aging in inflammatory skin diseases is often conceptualized as “inflammaging,” in which chronic inflammation acts as a primary determinant of advanced aging [31]. Recent transcriptomic and clinical studies have indeed suggested a strong association between AD and accelerated aging. For example, the impact of AD on epidermal barrier function has been shown to vary with age [32], and specific aging patterns in subjects with AD have been demonstrated through facial imaging analyses [33]. Furthermore, molecular evidence from recent studies investigating transcriptional age acceleration in inflammatory skin diseases supports this phenomenon [34]. Our finding that subjects with moderate AD tended to appear older than those with mild AD aligns with the notion that the degree of inflammation may be linked to the progression of skin‐aging determinants. These clinical observations, combined with our data on inflammatory markers such as IL‐8 and MMPs, highlight the role of AD‐related inflammation in premature facial aging.

In the treatment of AD, skincare—in the form of washing, moisturizing, and protecting from sunlight—is important for controlling symptoms [11, 12]. In the present study, AD‐Infrequent skincare had a lower stratum corneum water content, higher TEWL, darker skin, higher melanin index, lower pairwise youthfulness score, and higher levels of IL‐8 and MMP‐9 in their cheek skin than the HC group. Conversely, the absence of any significant difference between the AD‐Frequent skincare and HC group indicated that the skin of subjects who cared for their skin had a condition more closely resembling that of HCs. AD‐No‐Sunscreen had significantly higher levels of MMP‐9 in the stratum corneum than the HC group, indicating the importance of using sunscreen to provide sun protection. These findings suggested that everyday skincare habits may suppress age‐related external changes in the skin of AD subjects with mild facial symptoms.

**FIGURE 6:**
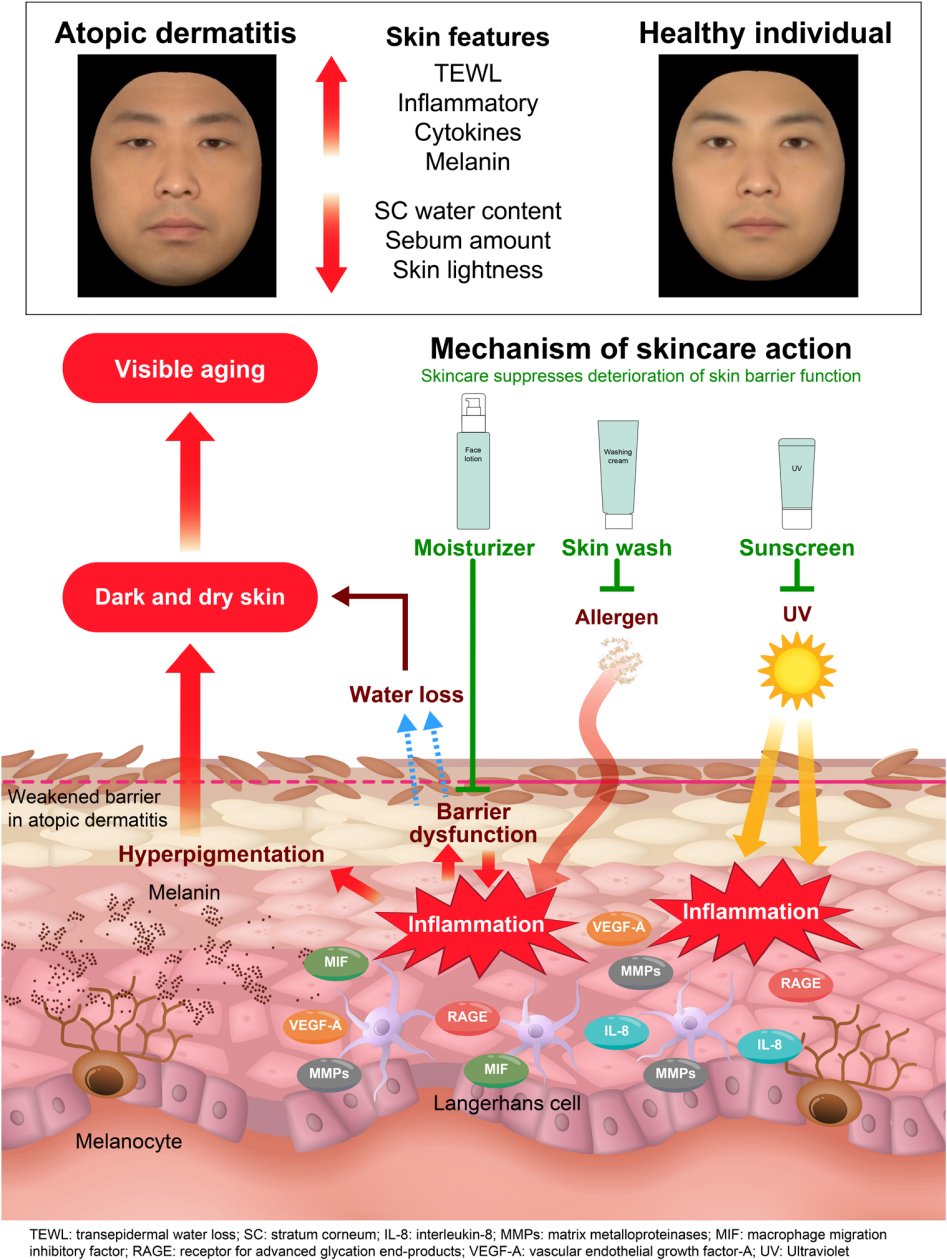
Skincare can suppress the acceleration of visible aging in subjects with atopic dermatitis. Subjects with atopic dermatitis exhibit dry and dull skin and barrier dysfunction. Although these factors contribute to visible aging, daily skincare can help slow its progression. SC, stratum corneum; TEWL, transepidermal water loss; UV, ultraviolet; IL, interleukin; MIF, macrophage migration inhibitory factor; MMP, matrix metalloproteinase; RAGE, receptor for advanced glycation end product; VEGF, vascular endothelial growth factor.

In the present study, we compared the faces of subjects with AD to those of HCs and showed that subjects with AD exhibit the age‐related skin changes—including skin dryness, barrier dysfunction, less sebum, and skin dullness—have progressed and appear older to a third party. We also demonstrated increased levels of physiological factors associated with inflammation in the stratum corneum of subjects with AD (Figure 6). Inflammation is regarded as a major cause of accelerated skin aging [10, 35], and the skin changes seen in subjects compared with HCs may have reflected the fact that their skin is continually inflamed. Although further in‐depth studies involving larger samples are required, our results in this study demonstrate that everyday skincare habits, including the use of sunscreen, may suppress age‐related skin changes in subjects with AD who have mild facial symptoms.

In this study, we compared the facial skin characteristics of subjects with mild AD with those of HCs. Future studies involving subjects with a wider range of AD severity are necessary to elucidate the effect of AD severity and further clarify the role of skincare habits in skin aging.

A limitation of this study is that the skincare habits and sunscreen use of the HC group were not characterized in the same detail as those of the AD group. Therefore, we cannot entirely rule out the possibility that differences in daily skincare routines between the two groups influenced the observed differences in physiological functions and visible aging. Regarding subgroup analyses and the resulting conclusions, we re‐evaluated our data to ensure that the statistical methods employed were appropriate for the sample sizes and distributions. Although our findings provide meaningful insights into the impact of skincare habits, further large‐scale studies with more rigorously matched backgrounds and broader statistical modeling are warranted to strengthen these conclusions.

## Author Contributions

Conception and design of the study: Katsuko Kikuchi, Yumi Murakami, and Hiroshi Matsunaka. Recruitment of subjects and acquisition of data: Haruna Sato, Ryoko Yamashita, Takuya Sugita, and Yosuke Shinkai. Analysis of data: Katsuko Kikuchi, Yumi Murakami, Haruna Sato, Yumiko Saya, Rikako Uchino, Takuya Sugita, Yosuke Shinkai, Yusuke Takahara, Kenta Shingaki. Drafting of the manuscript: Katsuko Kikuchi, Yumi Murakami, Takuya Sugita. Revising the manuscript critically for important intellectual content: Hiroshi Matsunaka. All authors reviewed and edited the manuscript and approved the final version of the manuscript.

## Funding

This study was supported by Tokiwa Pharmaceutical Co., Ltd.

## Ethics Statement

This study was approved by the Yoyogi Mental Clinic Research Ethics Committee (approval no. TWK312). A detailed explanation of the experimental procedure was provided to each participant before obtaining the signed consent form. All study participants provided informed consent to participate in this study. Written informed consent was obtained for anonymized patient information to be published in this article. The facial photographs included in this manuscript are synthesized images of the trial subjects' faces and are not individual facial images. Furthermore, the trial participants provided written consent for the use of their facial images following prior written and verbal explanations.

## Conflicts of Interest

This study was supported by TOKIWA Pharmaceutical Co. Ltd., but it was not conducted to evaluate or promote any specific products. The first author, Dr. Katsuko Kikuchi, is a physician affiliated with the Sendai Taihaku Dermatology Clinic and Department of Dermatology, Tohoku University Graduate School of Medicine, and the other authors are employees of TOKIWA Pharmaceutical Co. Ltd. or Noevir Co. Ltd. The study design, data analysis, and manuscript preparation were performed collaboratively while maintaining scientific neutrality. The authors declare no other conflicts of interest.

## Data Availability

The data that support the findings of this study are available from the corresponding author upon reasonable request.
